# Geochemical and geochronological dataset of rutile from a Variscan metabasite in Sardinia, Italy

**DOI:** 10.1016/j.dib.2020.105925

**Published:** 2020-06-25

**Authors:** Gabriele Cruciani, Dario Fancello, Marcello Franceschelli, Hans-Joachim Massonne, Antonio Langone, Massimo Scodina

**Affiliations:** a Dipartimento di Scienze Chimiche e Geologiche, Università di Cagliari - S.S. 554 Cittadella Universitaria 09042 Monserrato (CA), Italy; b School of Earth Sciences, China University of Geosciences, Lumo Road 388, 430074 Wuhan, China; c Istituto di Geoscienze e Georisorse-C.N.R. U.O.S. of Pavia, Via Ferrata 1, 27100 Pavia, Italy

**Keywords:** Rutile U/Pb ages, Rutile geochemistry, Variscan metabasite, Sardinia metamorphic basement

## Abstract

A c. 500 m wide and 1.5 km long body consisting of basic to ultrabasic rocks, metamorphosed up to granulite-facies and retrogressed to amphibolite-facies conditions during the Variscan orogeny, crops out near Olbia (NE Sardinia, Italy). Among abundant samples, one, collected from a garnet-rich centimetric layer, was chosen for a detailed analysis of rutile; chemical analyses of rutile were performed with the electron microprobe on petrographic thin sections, whereas U/Pb ages were determined by LA-ICP-MS on rutile mounted in epoxy resin. Chemical analyses show that rutile included in other minerals (Rt_inc_) commonly show higher SiO_2_ and FeO contents and lower Nb_2_O_3_ and ZrO_2_ contents if compared with rutile in the matrix of the garnet-rich layer (Rt_mat_). Cr_2_O_3_ concentrations are quite similar in both types of rutile. Rt_mat_ commonly shows a greater variability in minor elements, especially Nb_2_O_3_ (0.049–0.284 wt.%) and SiO_2_ (0.019 - 0.193 wt.%) whereas Rt_inc_ compositions are more homogeneous except for FeO (0.251–0.562 wt.%). The U-Pb isotopic data provided discordant ages and defined a lower intercept in the Tera-Wasserburg diagram of 273 ± 13 Ma. Few compilations of geochemical and geochronological data on rutile in Variscan metabasites can be found in literature, thus these data represent a new insight on a mineral phase the significance and scientific interest of which are rising in the last years. Future studies on the origin and ages of emplacement and metamorphism (either prograde or retrograde) of this kind of rock, widespread in the Variscan chain, will benefit from these data as a term of comparison.

Specifications table

**Table:** 

Subject	Earth and Planetary Sciences
Specific subject area	Geochemistry and petrology
Type of data	Table Chart Figure
How data were acquired	Field survey, sample collection, thin section and epoxy mounts Scanning Electron Microscope (SEM), FEI Quanta 200 coupled to Thermo Scientific™ UltraDry EDS Detector Electron MicroProbe (EMP), CAMECA SX100 193 nm ArF excimer laser ablation, GeoLas200Q-Microlas coupled to HR-ICP-MS, Thermo Finnigan Element I Software: Glitter
Data format	Raw and Analyzed Data
Parameters for data collection	Several samples of a Variscan metabasite were collected and the most representative one was chosen for these analyses. Thin sections were studied by optical and electron microscopy. Rutile was obtained by magnetic and heavy liquid separation after crushing the sample. The single grains were hand-picked and mounted in epoxy resin.
Description of data collection	SEM imaging was performed on polished and C-coated thin section Minerochemical data were acquired on polished and C-coated thin section by EMP U/Pb dating was performed by HR-ICP-MS-LA on rutile mounted in epoxy resin
Data source location	Olbia, Sardinia, Italy GPS coordinates: 9°34′29.7′’ E - 40°57′54.8′’ N (WGS84)
Data accessibility	With the article
Related research article	Some of the chemical analyses are listed in the supplementary material of the following: [1] M. Scodina, G. Cruciani, M. Franceschelli, H.-J. Massonne, Anticlockwise P-T evolution of amphibolites from NE Sardinia, Italy: Geodynamic implications for the tectonic evolution of the Variscan Corsica-Sardinia block, Lithos 324–325 (2019) 763–775. https://doi.org/10.1016/j.lithos.2018.12.003

## Value of the data

• Rutile provides constraints on the genesis, metamorphism and age of the host rock [2,3]
• Petrologists dealing with the evolution of the Variscan chain can found interesting information on rutile chemistry and ages
• Regional- scale studies could benefit from these data for comparison with similar rocks
• Few data about rutile in Variscan metabasites are available in literature
• The petrological interest for Ti-bearing mineral phases is growing in the last years [2]

## 1 Data description

### 1.1 Chemical analyses

**Fig. 1:**
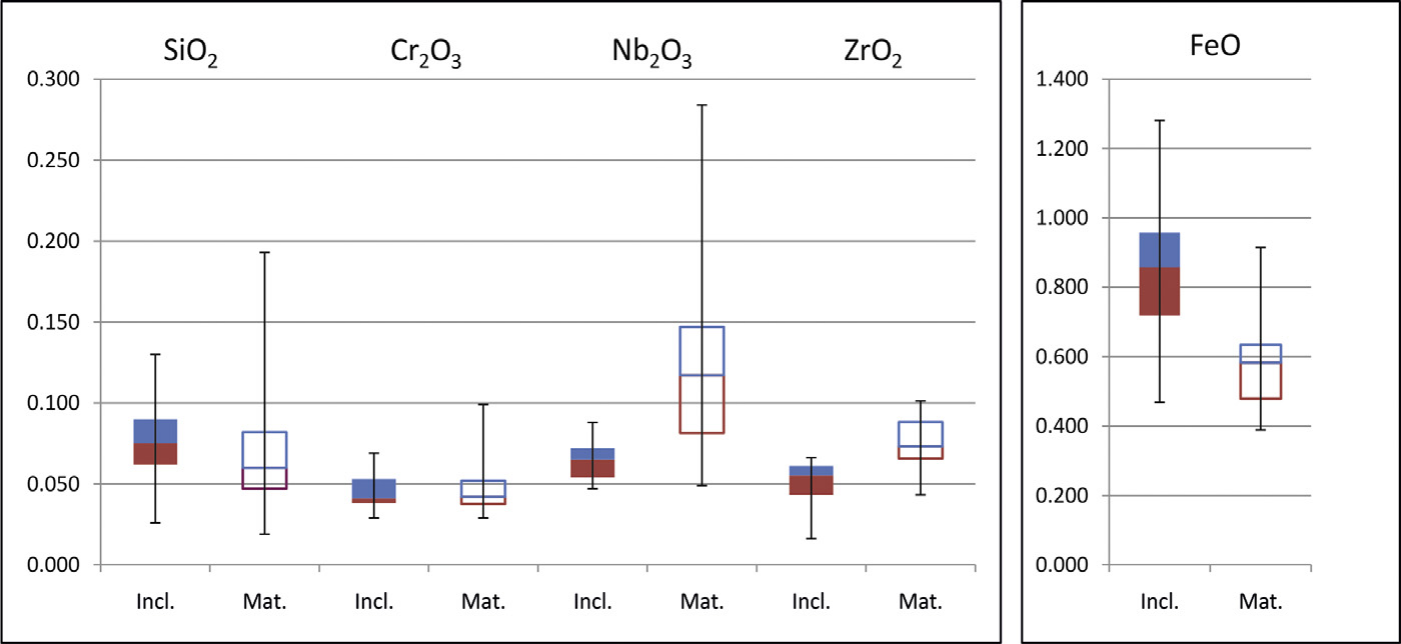
Box and whisker plots of major oxides (other than TiO_2_) analyzed in rutile either in the matrix (empty boxes) or as inclusions in other minerals (full boxes). FeO is displayed in a separate plot due to the different scale of the Y axis.

Chemical analyses of “major” oxides of *in-situ* rutile were determined by EMP on petrographic thin sections, allowing us to distinguish between rutile in the rock matrix (hereafter Rt_mat_) and as inclusions in other minerals (Rt_inc_) (Table 1 and 2, respectively, and Fig. 1). Part of these data (mainly Rt_inc_) belongs to the supplementary material of [1].

**Table 1:** EMP chemical analyses of rutile grains in the rock matrix (Rt_mat_) from thin section (*in-situ*). TiO_2_ was determined as difference from 100%.

		MN14a–Rutile in matrix						
Analysis n°		SiO_2_	TiO_2_	FeO	Cr_2_O_3_	Nb_2_O_3_	ZrO_2_	Total
*Rt mat. 1*	*	0.053	99.127	0.439	0.075	0.226	0.073	99.99
*Rt mat. 2*	*	0.064	99.109	0.441	0.075	0.230	0.076	100.00
*Rt mat. 3*	*	0.058	99.104	0.446	0.075	0.237	0.076	100.00
*Rt mat. 4*	*	0.045	99.100	0.607	0.032	0.141	0.068	99.99
*Rt mat. 5*	*	0.053	99.100	0.602	0.034	0.140	0.064	99.99
*Rt mat. 6*	*	0.047	99.077	0.624	0.035	0.141	0.070	99.99
*Rt mat. 7*	*	0.024	98.852	0.915	0.050	0.079	0.076	100.00
*Rt mat. 8*	*	0.019	99.344	0.428	0.051	0.075	0.074	99.99
*Rt mat. 9*	*	0.024	99.335	0.453	0.051	0.064	0.066	99.99
*Rt mat. 10*	⁎⁎	0.028	99.282	0.468	0.056	0.072	0.088	99.99
*Rt mat. 11*	⁎⁎	0.032	99.254	0.486	0.058	0.075	0.089	99.99
*Rt mat. 12*	⁎⁎	0.034	99.267	0.473	0.056	0.070	0.092	99.99
*Rt mat. 13*	⁎⁎	0.060	98.983	0.673	0.031	0.162	0.087	100.00
*Rt mat. 14*	⁎⁎	0.066	99.067	0.590	0.029	0.154	0.085	99.99
*Rt mat. 15*	⁎⁎	0.047	99.104	0.580	0.032	0.151	0.078	99.99
*Rt mat. 16*	⁎⁎	0.039	99.189	0.557	0.039	0.084	0.087	100.00
*Rt mat. 17*	⁎⁎	0.047	99.150	0.581	0.041	0.087	0.088	99.99
*Rt mat. 18*	⁎⁎	0.056	99.038	0.672	0.045	0.093	0.091	100.00
*Rt mat. 19*	⁎⁎	0.193	98.722	0.733	0.041	0.257	0.049	100.00
*Rt mat. 20*	⁎⁎	0.081	98.807	0.733	0.044	0.276	0.054	100.00
*Rt mat. 21*	⁎⁎	0.079	98.807	0.720	0.044	0.284	0.061	100.00
*Rt mat. 22*	⁎⁎	0.049	99.215	0.548	0.042	0.068	0.072	99.99
*Rt mat. 23*	⁎⁎	0.049	99.217	0.551	0.042	0.063	0.073	100.00
*Rt mat. 24*	⁎⁎	0.130	99.054	0.632	0.041	0.065	0.073	100.00
*Rt mat. 25*	⁎⁎	0.051	99.080	0.637	0.039	0.096	0.089	99.99
*Rt mat. 26*	⁎⁎	0.047	99.137	0.587	0.038	0.094	0.091	99.99
*Rt mat. 27*	⁎⁎	0.045	99.154	0.589	0.037	0.087	0.085	100.00
*Rt mat. 28*	⁎⁎	0.060	99.184	0.530	0.038	0.123	0.059	99.99
*Rt mat. 29*	⁎⁎	0.064	99.149	0.561	0.039	0.117	0.064	99.99
*Rt mat. 30*	⁎⁎	0.163	99.002	0.605	0.038	0.123	0.064	100.00
*Rt mat. 31*	⁎⁎	0.090	98.893	0.744	0.032	0.133	0.101	99.99
*Rt mat. 32*	⁎⁎	0.105	98.933	0.688	0.032	0.137	0.097	99.99
*Rt mat. 33*	⁎⁎	0.141	98.870	0.722	0.032	0.127	0.100	99.99
*Rt mat. 35*	⁎⁎	0.109	99.197	0.419	0.083	0.136	0.049	99.99
*Rt mat. 36*	⁎⁎	0.165	99.140	0.419	0.085	0.143	0.043	100.00
*Rt mat. 37*	⁎⁎	0.053	99.390	0.389	0.045	0.049	0.066	99.99
*Rt mat. 38*	⁎⁎	0.068	99.374	0.403	0.044	0.050	0.053	99.99
*Rt mat. 39*	⁎⁎	0.062	99.317	0.440	0.044	0.067	0.065	100.00
*Rt mat. 40*	⁎⁎	0.068	99.087	0.605	0.053	0.109	0.070	99.99
*Rt mat. 41*	⁎⁎	0.064	99.110	0.594	0.050	0.107	0.068	99.99
*Rt mat. 42*	⁎⁎	0.058	99.110	0.583	0.050	0.107	0.085	99.99
*Rt mat. 43*	⁎⁎	0.073	98.998	0.655	0.037	0.169	0.062	99.99
*Rt mat. 44*	⁎⁎	0.088	98.957	0.656	0.038	0.186	0.068	99.99
*Rt mat. 45*	⁎⁎	0.075	99.020	0.618	0.037	0.177	0.066	99.99
*Rt mat. 46*	⁎⁎	0.094	99.022	0.579	0.095	0.113	0.091	99.99
*Rt mat. 47*	⁎⁎	0.088	99.037	0.554	0.098	0.120	0.097	99.99
*Rt mat. 48*	⁎⁎	0.083	99.065	0.540	0.099	0.113	0.092	99.99

⁎ data from [1].

⁎⁎ data from this study.

**Table 2:** EMP Chemical analyses of rutile grains included in other minerals (Rt_inc_) from thin sections (*in-situ*). TiO_2_ was determined as difference from 100%.

		MN14a–Rutile inclusions						
Analysis n°		SiO_2_	TiO_2_	FeO	Cr_2_O_3_	Nb_2_O_3_	ZrO_2_	Total
*Rt inc. 1*	*	0.116	99.104	0.659	0.047	0.053	0.016	100.00
*Rt inc. 2*	*	0.075	99.239	0.553	0.050	0.058	0.019	99.99
*Rt inc. 3*	*	0.062	99.212	0.529	0.066	0.069	0.055	99.99
*Rt inc. 4*	*	0.053	99.240	0.507	0.069	0.072	0.053	99.99
*Rt inc. 5*	*	0.053	99.279	0.468	0.069	0.078	0.047	99.99
*Rt inc. 6*	*	0.062	98.835	0.946	0.038	0.069	0.043	99.99
*Rt inc. 7*	*	0.056	98.827	0.958	0.041	0.069	0.043	99.99
*Rt inc. 8*	*	0.062	98.822	0.957	0.039	0.072	0.043	100.00
*Rt inc. 9*	*	0.064	98.938	0.844	0.039	0.047	0.061	99.99
*Rt inc. 10*	*	0.081	98.887	0.876	0.038	0.052	0.061	100.00
*Rt inc. 11*	*	0.071	98.863	0.911	0.038	0.052	0.059	99.99
*Rt inc. 12*	*	0.051	99.094	0.665	0.038	0.083	0.062	99.99
*Rt inc. 13*	*	0.073	99.010	0.719	0.041	0.084	0.066	99.99
*Rt inc. 14*	*	0.073	99.010	0.736	0.039	0.082	0.055	100.00
*Rt inc. 15*	*	0.073	98.908	0.837	0.039	0.072	0.064	99.99
*Rt inc. 16*	*	0.090	98.832	0.892	0.039	0.081	0.062	100.00
*Rt inc. 17*	*	0.088	98.878	0.858	0.038	0.072	0.061	100.00
*Rt inc. 18*	*	0.068	98.968	0.785	0.034	0.082	0.055	99.99
*Rt inc. 19*	*	0.083	98.695	1.047	0.029	0.088	0.050	99.99
*Rt inc. 20*	*	0.090	99.065	0.682	0.037	0.065	0.055	99.99
*Rt inc. 21*	*	0.124	98.423	1.281	0.042	0.060	0.062	99.99
*Rt inc. 22*	*	0.130	98.561	1.139	0.044	0.057	0.061	99.99
*Rt inc. 23*	*	0.103	98.511	1.211	0.042	0.065	0.064	100.00
*Rt inc. 24*	*	0.083	98.872	0.898	0.058	0.053	0.030	99.99
*Rt inc. 25*	*	0.094	98.905	0.863	0.056	0.052	0.024	99.99
*Rt inc. 26*	*	0.088	98.942	0.822	0.053	0.060	0.027	99.99
*Rt inc. 33*	*	0.107	98.672	1.057	0.056	0.054	0.049	100.00
*Rt inc. 34*	*	0.096	98.667	1.069	0.058	0.054	0.047	99.99
*Rt inc. 38*	*	0.026	98.953	0.858	0.042	0.055	0.059	99.99

⁎ data from [1].

TiO_2_ content in Rt_inc_ ranges between 98.423 and 99.279 wt.% (avg. 98.908 wt.%), slightly lower than that of Rt_mat_ ranging 98.722 - 99.390 wt.% (avg. 99.104 wt.%). FeO, the main oxide in rutile other than TiO_2_, shows quite variable contents with a wider compositional range for Rt_inc_ (0.468 - 1.281 wt.%) than for Rt_mat_ (0.389–0.915 wt.%). The interquartile range (IQR) better shows the different FeO concentration in the two rutile populations; Rt_inc_ are commonly richer in iron (IQR = 0.72–0.96 wt.%) than Rt_mat_ (IQR = 0.48–0.64 wt.%). Silica contents in Rt_inc_ and Rt_mat_ are comparable considering the IQR (0.062–0.090 and 0.047–0.082, respectively) whereas, looking at the whole range, a high data dispersion is observed with values up to 0.193 wt.% in Rt_mat_. Cr_2_O_3_ contents are quite similar in the two rutile populations showing IQR 0.038–0.053 wt.% for both types even if Rt_mat_ reaches values up to 0.099 wt.%. Rt_mat_ hosts higher amounts of Nb_2_O_3_ (IQR = 0.082–0.147 wt.%) and ZrO_2_ (IQR = 0.066–0.088 wt.%) if compared with Rt_inc_ (0.054–0.072 and 0.043–0.061 wt.%, respectively). It is worth of note the high variability of Nb_2_O_3_ in Rt_mat_ that shows concentrations up to 0.284 wt.%, three times the maximum value in Rt_inc_.

Additional analyses of trace elements were determined by LA-ICP-MS, on rutile in a resin mount (*ex-situ* rutile*,* Table 3). The concentrations of Nb (510–1360 ppm), Zr (422–560 ppm) and Cr (164–369 ppm) of such rutile, converted to wt.% of oxides (Nb_2_O_3_ 0.073–0.195; ZrO_2_ 0.057–0.076; Cr_2_O_3_ 0.024–0.054 wt.%), are well comparable to those determined on *in-situ* rutile by EMP, testifying the reliability of LA-ICP-MS analyses. In particular, Zr and Nb suggest that *ex-situ* rutile was hosted in the rock matrix (instead of being inclusions in other minerals) since their concentration match quite well those determined on Rt_mat_. LA-ICP-MS analyses also reveal that V is the most abundant trace element, being in almost all analyzed grains > 1000 ppm. Subordinate trace elements are Zn ranging between 2.6 and 21.7 ppm, Sr 2.8–3.8 ppm, Ta 14–42 ppm, Hf 12–16 ppm and U 9–19 ppm. Many of the other trace elements including Mn, Ni, Rb and all REE (light, medium and heavy) are below detection limit for most of the analyzed rutiles.

**Table 3:** LA-ICP-MS analyses of trace elements of rutile from a resin mount (*ex-situ*).

	MN14a–*ex-situ* rutiles								
	*Rt3*	*Rt4*	*Rt6*	*Rt7*	*Rt8*	*Rt14*	*Rt15*	*Rt16*	*Rt17*
ppm									
Sc	1.85	1.83	1.35	2.01	2.02	1.69	1.99	1.63	1.58
V	1042.89	922.96	1236.69	1359.99	1035.51	1031.80	1035.83	959.00	1068.78
Cr	164.41	196.67	295.72	308.15	368.83	252.98	269.64	211.63	263.78
Co	0.011	0.023	0.057	0.036	0.049	0.029	0.055	bdl	bdl
Cu	1.441	0.995	0.924	1.595	1.029	1.186	1.118	1.042	3.28
Zn	21.70	11.33	7.46	14.23	5.84	4.69	6.08	12.20	2.60
Sr	3.083	3.420	3.158	3.800	3.400	2.762	3.510	2.985	3.510
Y	0.228	0.234	0.149	0.279	0.253	0.245	0.185	0.198	0.150
Zr	559.92	464.97	483.68	523.41	495.17	443.94	545.18	421.63	494.06
Nb	664.24	907.74	555.88	509.85	1360.15	715.34	609.28	715.68	511.10
Mo	34.20	61.50	27.47	23.25	77.07	34.74	33.35	73.29	26.57
In	0.145	0.136	0.133	0.170	0.157	0.066	0.185	0.084	0.126
Ba	0.030	0.196	0.111	0.640	0.028	0.005	0.037	0.013	bdl
Hf	16.51	13.33	13.08	14.22	14.27	14.25	14.40	12.15	12.01
Ta	13.84	32.31	20.48	23.48	40.98	31.74	32.06	41.56	22.91
Pb	0.039	bdl	bdl	0.101	0.062	bdl	0.061	bdl	bdl
U	18.84	8.79	9.27	13.32	10.85	9.90	9.58	14.32	9.87

All data from this study. bdl = below detection limit.

### 1.2 U/Pb radiometric dating

U/Pb analyses were performed on rutile embedded in an epoxy resin mount. Thus, is not possible to assess whether or not Rt_mat_ and Rt_inc_ record different radiometric ages. Analyzed rutile is commonly homogeneous, free of inclusions and euhedral with shallow to pervasive fractures. The shape is mainly elongated and the size ranges between 100 and 150 µm in length and 40–60 µm in width. Subhedral and rounded grains are also found, probably resulting from intense fracturing. Rare ilmenite exsolutions, as thin needles, are found in rutile.

13 points in as many rutile grains were analyzed but 4 analyses were rejected due to a low U signal; results are listed in Table 4.

**Table 4:** LA-ICP-MS U–Pb results for rutile from metabasite sample MN14a.

	Isotopic ratios							Ages						
Point	^207^Pb/^206^Pb	1 s%	^207^Pb/^235^U	1 s%	^206^Pb/^238^U	1 s%	ρ	^207^Pb/^206^Pb	1 s abs	^207^Pb/^235^U	1 s abs	^206^Pb/^238^U	1 s abs	% disc
*Rt 05*	0.0646	0.0017	0.4029	0.0193	0.0452	0.0014	0.6309	760	20	344	16	285	9	17
*Rt 06*	0.1242	0.0032	0.7501	0.0359	0.0437	0.0013	0.6333	2017	53	568	27	276	8	51
*Rt 07*	0.1246	0.0034	0.7834	0.0379	0.0450	0.0014	0.6336	2023	55	587	28	284	9	52
*Rt 08*	0.0927	0.0025	0.5813	0.0281	0.0453	0.0014	0.6293	1481	40	465	22	286	9	39
*Rt 10*	0.0962	0.0025	0.6025	0.0290	0.0454	0.0014	0.6294	1551	41	479	23	286	9	40
*Rt 12*	0.0966	0.0026	0.6006	0.0290	0.0446	0.0014	0.6325	1559	42	478	23	281	9	41
*Rt 13*	0.1348	0.0035	0.8619	0.0412	0.0463	0.0014	0.6345	2161	56	631	30	292	9	54
*Rt 14*	0.1090	0.0029	0.6965	0.0335	0.0463	0.0014	0.6309	1783	47	537	26	292	9	46
*Rt 15*	0.0776	0.0020	0.4519	0.0216	0.0416	0.0013	0.6354	1137	29	379	18	263	8	31

**Fig. 2:**
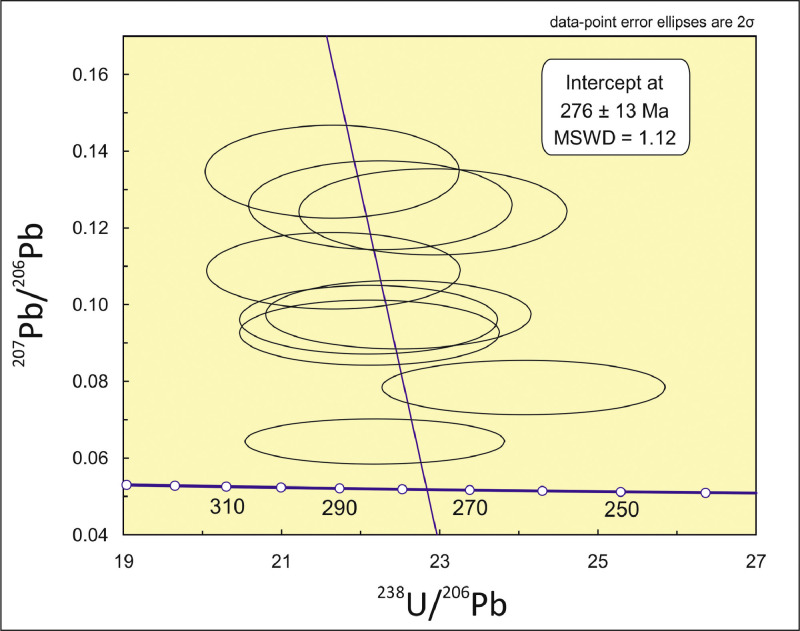
Tera-Wasserburg diagram [4] showing isotopic data and the lower intercept age of the analyzed rutile.

The analyzed rutile gave discordant ^206^Pb/^238^U and ^207^Pb/^235^U ages. In the Tera-Wasserburg plot [4] (Fig. 2) the Pb uncorrected rutile data define a lower intercept at 276 ± 13 Ma.

## 2 Experimental design, materials, and methods

### 2.1 Geological setting

**Fig. 3:**
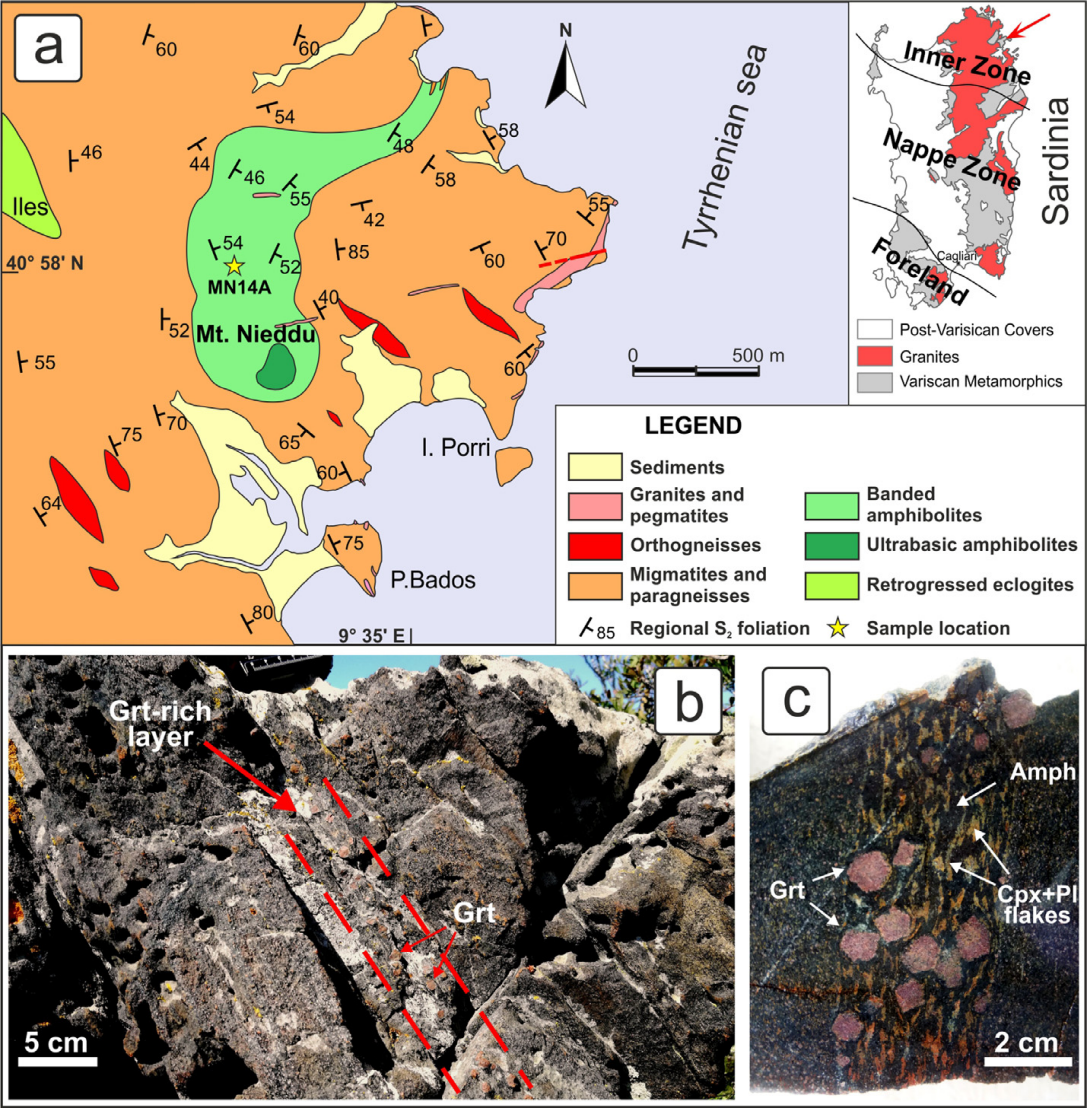
a) Geological map of the area surrounding the metabasite body, the location of which is highlighted by the red arrow in the inlet showing a simplified tectonic map of Sardinia; b) outcrop photograph of the garnet-rich layer; c) detail of the garnet-rich layer on a cut and polished hand-sample.

The Sardinian metamorphic basement is a segment of the Variscan orogenic belt, showing an almost complete transect of the chain, from the Foreland in the southwest to the Inner (or Axial) Zone in the north-northeast [5]. Two different metamorphic complexes, separated by a WNW-ESE-trending shear zone named Posada Asinara Line (PAL) [6], have been distinguished in the Inner Zone: the High-Grade Metamorphic Complex (HGMC, or Migmatite Complex) north of the PAL and the Medium-Grade Metamorphic Complex (MGMC), south of the PAL (Fig. 3a).

The HGMC is mainly made up by gneiss and migmatites [7,8,9] that record a multi-stage deformation history in which at least three main phases have been recognized [10,11]. The occurrence of marble [12], calcsilicate rocks and especially metabasite lenses and bodies [13,14] is quite common. Metabasites are very interesting because, although retrogressed to amphibolite/greenschist-facies conditions, they still preserve relics of the granulite and eclogite facies [1,14,15].

The largest metabasite body in the HGMC crops out NE of Olbia in the Montiggiu Nieddu locality (hereafter Mt. Nieddu) and consists of a 1.5 km long and 350 to 500 m wide body roughly elongated in SW-NE direction. The main lithology consists of a banded amphibolite formed by alternating centimeter- to decimeter-thick whitish plagioclase-rich bands and dark green amphibole-rich bands; in addition, epidote-rich veins and centimeter-thick levels hosting abundant garnet grains, up to 1 cm in size, are locally found within the banded amphibolite [1,16] (Fig. 3b,c). On the southern side of Mt. Nieddu, a smaller body of metabasite, derived from an ultrabasic igneous protolith, occurs; it preserves relics of igneous structures and minerals, as well as relics of the granulite facies [17].

The here presented data come from a sample of a garnet-rich layer collected in the banded amphibolite; such layers have been interpreted as the less retrogressed rocks of the Mt. Nieddu complex and thus are suitable to reconstruct the early metamorphic stages.

### 2.2 Petrography

**Fig. 4:**
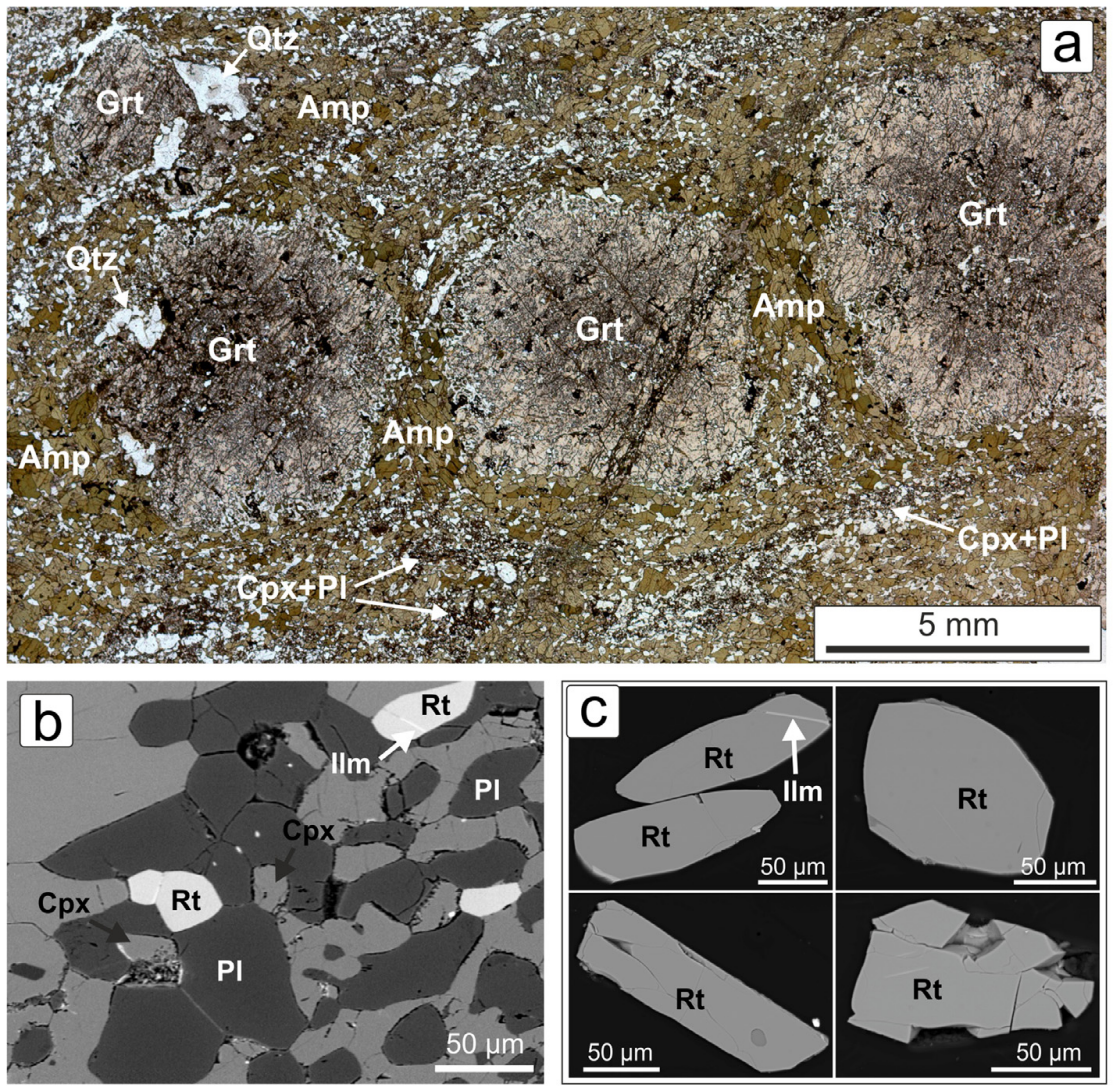
a) Photomicrograph of the MN14a thin section under plane polarized light; b) BSE image of the same sample; c) different occurrences of rutile from euhedral to anhedral, from fresh to strongly fractured grains, seldom with ilmenite exsolutions. Mineral abbreviations: quartz (Qtz), amphibole (Amp), garnet (Grt), clinopyroxene (Cpx), plagioclase (Pl), rutile (Rt), ilmenite (Ilm).

The garnet-rich layer is characterized by abundant (up to 30% vol.) sub-centimetric garnet porphyroblasts in a matrix consisting of amphibole, plagioclase, quartz andclinopyroxene + plagioclase symplectite. Garnet is rimmed by sub-millimetric coronas of plagioclase + amphibole and hosts inclusions of amphibole, plagioclase, quartz, clinopyroxene, rutile and ilmenite (Fig. 4a). Accessory minerals are rutile, titanite, zircon, K-feldspar, chlorite, Fe-oxides, monazite, and biotite. Rutile grains occur in both garnet and matrix and are mainly free of inclusions. These grains are euhedral with elongated shape and length ranging between 80–130 µm for Rt_mat_ and 20–30 µm for Rt_inc_. Subhedral and rounded grains are also found, probably resulting from intense fracturing. Ilmenite replacement and thin titanite rims rarely occur (Fig. 4b,c).

### 2.3 Materials and methods

Polished thin sections were prepared from a selected sample of the garnet-rich layer. The preliminary observation, under a polarization microscope, was followed by BSE imaging performed at CeSAR (Centro Servizi d'Ateneo per la Ricerca, Università di Cagliari) with a scanning electron microscope FEI Quanta 200, equipped with a Thermo Scientific™ UltraDry EDS Detector for qualitative minero-chemical analyses. The quantitative chemical analyses of the here reported rutile were performed with a CAMECA SX100 electron microprobe (EMP) equipped with five wave-length dispersive (WD) spectrometers at the (former) Institut für Mineralogie und Kristallchemie, Universität Stuttgart. The operative conditions were 15 kV acceleration voltage, 200 nA beam current and 5 μm spot size. For the description of the used standards, counting times and analytical errors see [18], [19]. SEM and EMP analyses were performed on thin sections (i.e.*in-situ*) allowing us to distinguish between rutile inclusions in other minerals and rutile in the rock matrix.

On the contrary, *ex-situ* rutile embedded in a resin mount was used for U/Pb radiometric dating performed at the CNR-Istituto di Geoscienze e Georisorse (Pavia, Italy) by ablation with a 193 nm ArF excimer laser (GeoLas200Q-Microlas) coupled to an Agilent 8900 quadrupole mass spectrometer. About 4 kg sample material was carefully crushed. Rutile crystals were separated by magnetic and heavy liquid separation. Finally, the single grains were hand-picked and mounted in epoxy resin. The rutile-bearing mount was then polished to reach the core of the grains. The external reproducibility was calculated from the standard Sugluk-4 and PCA-S207 [20] analyzed during the analytical session whereas the errors were propagated according to [21].

## Declaration of Competing Interest

The authors declare that they have no known competing financial interests or personal relationships which have, or could be perceived to have, influenced the work reported in this article.
